# The Role of Cell Tracing and Fate Mapping Experiments in Cardiac Outflow Tract Development, New Opportunities through Emerging Technologies

**DOI:** 10.3390/jcdd8050047

**Published:** 2021-04-26

**Authors:** Joshua C. Peterson, Tim P. Kelder, Marie José T. H. Goumans, Monique R. M. Jongbloed, Marco C. DeRuiter

**Affiliations:** 1 Department Anatomy & Embryology, Leiden University Medical Center, 2300RC Leiden, The Netherlands; J.C.Peterson@lumc.nl (J.C.P.); T.P.Kelder@lumc.nl (T.P.K.); M.R.M.Jongbloed@lumc.nl (M.R.M.J.); 2 Department Cellular and Chemical Biology, Leiden University Medical Center, 2300RC Leiden, The Netherlands; M.J.T.H.Goumans@lumc.nl; 3 Department of Cardiology, Leiden University Medical Center, 2300RC Leiden, The Netherlands

**Keywords:** cardiac development, congenital heart disease, lineage tracing, outflow tract, developmental biology, bicuspid aortic valve, cell identity

## Abstract

Whilst knowledge regarding the pathophysiology of congenital heart disease (CHDs) has advanced greatly in recent years, the underlying developmental processes affecting the cardiac outflow tract (OFT) such as bicuspid aortic valve, tetralogy of Fallot and transposition of the great arteries remain poorly understood. Common among CHDs affecting the OFT, is a large variation in disease phenotypes. Even though the different cell lineages contributing to OFT development have been studied for many decades, it remains challenging to relate cell lineage dynamics to the morphologic variation observed in OFT pathologies. We postulate that the variation observed in cellular contribution in these congenital heart diseases might be related to underlying cell lineage dynamics of which little is known. We believe this gap in knowledge is mainly the result of technical limitations in experimental methods used for cell lineage analysis. The aim of this review is to provide an overview of historical fate mapping and cell tracing techniques used to study OFT development and introduce emerging technologies which provide new opportunities that will aid our understanding of the cellular dynamics underlying OFT pathology.

## 1. General Introduction

The development of the cardiac outflow tract (OFT), a region of the heart connecting the ventricles to the great arteries of the heart, has been studied for more than a century [1]. Cellular lineage tracing experiments laid the foundation for our knowledge regarding congenital heart disease (CHD). Whilst major progress has been made and CHDs as a whole are understood increasingly better, the degree of understanding varies greatly among the individual congenital defects. CHDs affecting the OFT like bicuspid aortic valve, tetralogy of Fallot, transposition of the great arteries, double outlet right ventricle and pulmonary atresia are well known disorders, yet it remains challenging to explain such morphological emergences with our current models of OFT development. Common among many of these congenital diseases is a broad phenotypical variation arising from a singular genetic deficiency. Murine knockout models used to study such aforementioned CHDs, such as *Notch1* [2], *Gata5* [3] and *Nos3* [4] mutant mice, have been known for their incomplete penetration, yet any agreement addressing such phenotypical variation remains elusive. Whilst lineage tracing studies have contributed significantly to our understanding of a particular cell type heritage, it still falls short to explain, how a cell’s lineage primes cellular behavior to allow for proper cell adaptation (such as cell maturation) upon environmental cues. Epigenetic modification of chromatin have been described to affect gene expression and could act to consolidate directional developmental gradients [5,6]. As such a cell’s identity may arise not only from a genetic component but from a combination of expressed genes and cell lineage epigenetic signatures [7]. 

A single genetic mutation could give rise to multiple phenotypical outcomes if the imprinted epigenetics are dissimilar among cell lineages [8]. Currently we are limited in our understanding about the role of cell identity in CHD. However given that a cell’s identity is highly plastic [9], understanding the role of the identity of a cell might explain the broad phenotypical variation observed in these congenital heart diseases and could aid with disease risk stratification. Lineage tracing experiments built the foundation of our current understanding and new developments in methods could provide more detailed definitions of cell types and cell lineage dynamics to better explain how aberrant cell behavior contributes to the multivariate phenotypes seen in CHD.

### 1.1. Basic Cardiac Development and Anatomy of the OFT

The heart is formed from the anterior splanchnic mesoderm of the embryonic plate. During gastrulation, mesodermal cells arise from the primitive streak, and migrate cranially and laterally to the notochord to form the cardiogenic plates expressing Nkx2.5 [10] and GATA4 [11]. The first sign of cardiomyocyte differentiation is seen in this region at Hamburger and Hamilton (HH) stage 8–9 in chick and between embryonic day (E) 6.5–7.5 in mice, when cardiac troponin-I (cTnI) and sarcomeric myosin (MF20) are first detected. Fusion of the bilateral plates of splanchnic mesoderm establishes the primary heart tube (PHT), that shows peristaltic contraction at 3 weeks of development in a human, E8.0–8.5 in mouse and HH stage 10–11 in chick embryos [12,13,14,15]. During further development, cells from the splanchnic mesoderm differentiate into cardiomyocytes and are added to the PHT. The splanchnic mesoderm that gives rise to the cardiomyocytes of the cardiac crescent and PHT is called the first heart field (FHF). The FHF will contribute to part of the atria, atrioventricular canal (AVC), inlet of the right ventricle and left ventricle [12]. In the next phase of heart formation, cells are added to specific structures at the venous (atrial septum and smooth myocardial wall of the atrium) and arterial poles (outflow tract and the right ventricle). These cells are considered to be derived from a distinct cardiogenic field, the second heart field (SHF) [12]. How the distinction between FHF and SHF is related to the ultimate phenotype of the left and right ventricular myocardium is an intriguing question but is not the focus of this review. The addition of cells at the arterial and venous poles initiates the process of cardiac looping, the transformation of the linear heart tube into an S-shaped heart tube with primitive ventricular and atrial chambers [16]. These primitive chambers are connected by the AVC. In the AVC and the OFT, the myocardium secretes cardiac jelly, a hydrophilic substance rich in hyaluronic acid, proteoglycans and extracellular matrix. This results in the formation of localised swellings called the endocardial cushions acting as primitive valves, aiding in maintaining unidirectional flow in the heart tube [17,18]. The endocardial cushions of the OFT mature into aortic and pulmonary valves through contribution of cell lineages of neural crest [19,20], endothelial [21,22] and second heart field cells [23,24,25], but are also crucial to the development of the interventricular septum [18,26]. The separation of the aorta and the pulmonary trunk is the result of OFT septation. During OFT septation, extracardiac neural crest cells migrate into the endocardial cushions forming a central mass of condensed mesenchyme, resulting in an aortopulmonary septum followed by invagination of cardiomyocytes. The rotation and elongation of the aortic and pulmonary orifices is the result of the pulmonary push driven by cells of the second heart field lineage [27]. OFT septation is completed when the aortic orifice connects to the left ventricular outflow tract whilst the pulmonary orifice remains connected above the right ventricle [28]. In addition to cardiac growth, several processes, including the outgrowth and remodelling of the different chambers, coronary artery development and formation of the cardiac conduction system, will establish the mature electro-mechanically functional 4-chambered heart with separated pulmonary and systemic circulations (reviewed in [29,30]).

### 1.2. OFT Development

The OFT can first be observed between E8.0–E8.5 in mice and week 3 of development in humans during formation of the PHT where it connects the primitive ventricle with the aortic sac. During cardiac looping the OFT elongates and remodels forming the OFT cushions. Aortopulmonary septation occurs at the level of: (1) the great arteries, (2) the valvular level and (3) of the outflow tract [31]. 

The aortopulmonary septum is formed by contributions of neural crest cells and second heart field cells [32,33,34]. The initially unseparated vascular part of the outflow tract is called the aortic sac. For adequate separation of the different levels of the developing outflow tract, development of the aortopulmonary septum and adequate fusion of the outflow cushions are mandatory [35]. Neural crest cells will be positioned at the level of the aortic sac, as well as in the condensed mesenchyme of the septal outflow tract cushion at and below the orifice level [36]. As a result of this process, separation of the aortic sac will extend from the arterial orifice level (i.e., the level of the developing arterial valve) to the myocardial outflow tract.

The aortopulmonary septum will gradually separate the common aortopulmonary trunk into the aorta and pulmonary trunk. At the valvular level, this process will also separate the OFT cushions at the aortic and pulmonary orifices giving rise to the semilunar valves. Later the coronary arteries will grow into the aortic sinuses of Valsalva and the myocardial and smooth muscle components of the OFT will mature. 

Historically, the developing OFT has been studied using classical light microscopy, histological staining and electron microscopy [14,37,38]. A comprehensive basis providing insight in OFT development was founded using these techniques. Unfortunately, as cells undergo intrinsic and phenotypical changes during development, analysing the developmental origin of the OFT was not possible using these early methods. To examine cell lineage development, several techniques such as genetic tracing and vital dye labelling of cells have been developed. Extensive lineage tracing experiments examining OFT morphogenesis have revealed an important role for multiple cell types and signaling pathways in OFT development. 

## 2. Cell Tracing Techniques Applied to OFT Development

In the next section, the different techniques used to study OFT development will be described briefly. The main focus of this section will be to review fate mapping experiments that contributed to our current understanding of OFT development.

### 2.1. Vital Dye and Viral Labelling Experiments

With vital dye it is possible to directly label clusters of cells. This method is widely used in developmental biology in several species and has provided essential information on the developmental origin of the OFT.

#### 2.1.1. Basics of Vital Dye and Viral Labelling Experiments during Heart Development

Labelling of cells is performed with a glass capillary mounted to micromanipulators, and a pneumatic micro-injector to inject small amounts of dye or virus in the region of interest. 

**Figure 1:**
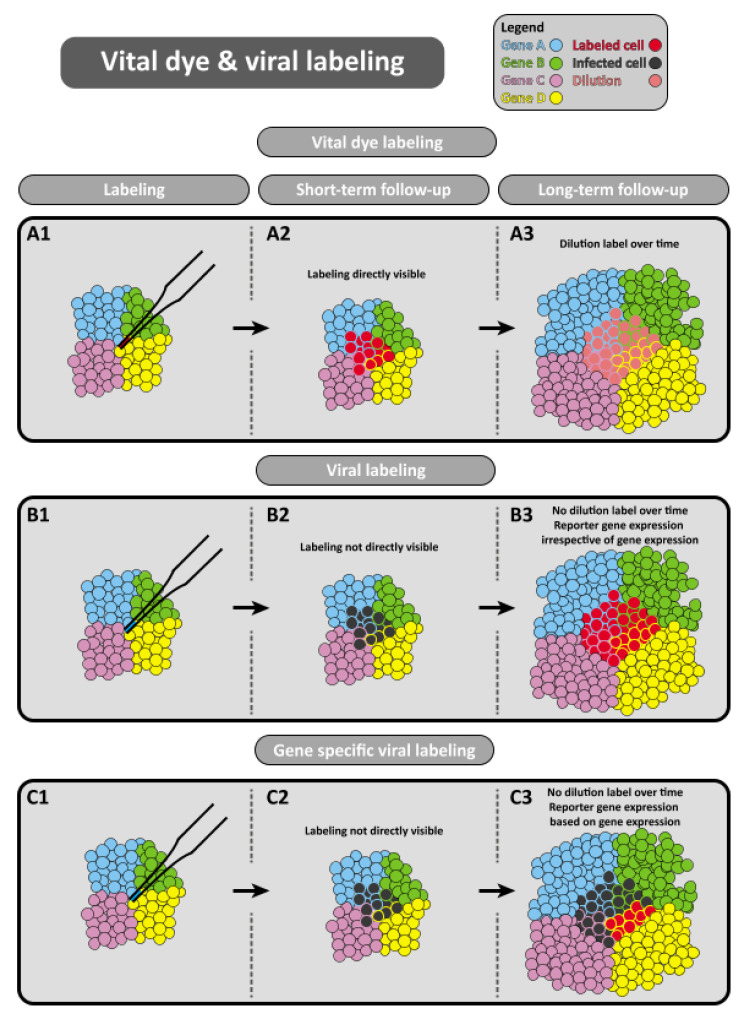
Vital dye and viral labeling techniques. (**A**) Lipophilic dyes are used to visualise and track distinct cell compartments over time. (**B**) Alternatively, retroviral vectors can allow for expression of a reporter gene (such as *LacZ*) to enable cell tracing of infected cells. (**C**) Placing the reporter gene under the transcriptional control of a cell specific promoter allows for increased accuracy in targeting cells through gene specific viral labelling.

Vital dye labelling is performed with lipophilic dyes, such as diI-C18-(3) [39], which can label distinct cell compartments (Figure 1A) [40]. Commercial dyes are available in different colours, which enables simultaneous labelling of different clusters of cells and studying the direction of migration and potential intermingling of different cell clusters. 

Viral labelling experiments are aimed at induction of reporter gene expression in specific regions of the embryo. This technique utilises the ability of retroviruses to infect the cell and to incorporate its genome in the genome of the infected cell [41,42,43]. Retroviral vectors driving the expression of a reporter gene (such as *LacZ*) enable cell tracing of the infected cells (Figure 1B). More targeted viral tracing can be performed by placing expression of the reporter gene under the transcriptional control of a cell type specific promoter, which enables tracing of cells that express the gene of interest (Figure 1C) [44]. 

#### 2.1.2. Advantages of Vital Dye Labelling Experiments

With physical labelling of a progenitor pool, it is possible to select that specific group of cells based on location and to time the exact moment of labelling. An important advantage of this technique is the opportunity to select a cluster of cells based without prior knowledge of the cell type. Performing physical labelling experiments is relatively cost efficient and easy, especially in chick, fish and amphibian embryos. Furthermore, labelling can be combined with other microsurgical procedures. 

#### 2.1.3. Limitations of Vital Dye and Viral Labelling

To perform microinjection of minute quantities of either vital dye or viral constructs, the embryo needs to be physically accessible to the researcher. For this reason most labelling experiments examining heart development using micro-injections have been performed in oviparous vertebrates, such as zebrafish [45], xenopus [46] and chick embryos [47,48]. For use in mice, it is first necessary to extract the embryo from the uterus, followed by re-incubation in culture medium, for example in rolling bottles [49].

Physical labelling experiments require a progenitor population of interest to study. Knowledge about the location of a possible progenitor pool is required to correctly target these cells. Moreover, when targeting a progenitor population, the lineage trace is limited to cells solely derived from that original cluster. This complicates examining structures derived from multiple progenitor clusters because non-labelled cell contributions could be interpreted as inefficient labelling. 

Furthermore, physically labelling cells is relatively invasive, and manipulation of the embryo may induce developmental defects. It is therefore necessary to carefully select embryos that show no macroscopic abnormalities and to include control embryos. Toxicity of the dyes itself appears to be negligible [50,51]. 

With viral labelling, there is random integration of the viral genome in the host cell’s DNA, which could result in damage or abnormal functioning of the infected cell [52]. Leakage of dye or infection of cells in the vicinity of the designated progenitor pool could result in incorrect interpretation regarding the fate of the progenitor cell population. It is therefore essential to evaluate the location of dye directly after administration in great detail [40,53]. Histological evaluation of embryos directly after labelling is crucial, but this also terminates lineage tracing in those embryos. Live-imaging of vital dyes [51,54,55] could help to minimise this limitation. Furthermore, delay in reporter gene expression with viral labelling omits direct evaluation of the location of labelling. In addition, efficiency of reporter gene expression in the desired cells has to be tested prior to ascertain activation of expression in the specific cell type within the animal model (i.e., species differences in expression levels in specific cell types). Rapid cell division in early embryos results in dilution of the vital dyes, which complicates long-term follow-up of the labelled cells [56]. The extent of dilution is dependent on the rate of proliferation and the amount of dye administered, which therefore necessitates titration of the dye with every new experiment. With retroviral labelling, integration of the reporter gene in the host cell’s DNA results in stable and high expression of the reporter gene in the daughter cells [57], which overcomes the problem of dilution. Therefore, a disadvantage of vital dye labelling is dilution, whilst the possibility of direct evaluation after labelling is an important advantage. The opposite is true for viral labelling, where direct evaluation is not possible, but dilution of dye does not occur after stable integration of the viral vector. Therefore, a possible solution to overcome these limitations is to combine vital dye labelling with viral labelling. Finally, to perform viral labelling experiments, advanced biosafety laboratories are crucial to safely perform experiments with potentially hazardous viral constructs. 

#### 2.1.4. Vital Dye and Viral Labelling Experiments Aimed at Understanding OFT Development

Studies performing dye injected lineage tracing have aided in understanding heart development by carefully fate mapping groups of cardiac precursor cells [58,59]. Prior to the concept of vital dye lineage tracing descriptive embryological studies in the early 20th century examining human embryos postulated the concept of the primitive cardiac cavities [38]. These primitive cardiac cavities where demarcated by local folds. Using these folds as fixed borders, the outgrowth of cardiac structures such as the development of the atria and ventricles could be described. The introduction of vital dye injected lineage tracing pioneered by de la Cruz refuted the concept of primitive cardiac cavities and showed that structures such as the primordium of the trabeculated portion of the right ventricle could be determined at the straight heart tube stage prior to the formation of primitive cardiac cavities [47]. 

### 2.2. Lineage Tracing Using the Quail-Chick Chimeric System

The use of chimeric models is an alternative lineage tracing approach as compared to the previous mentioned vital dye injections. The quail-chick chimeric system proved a vital tool for understanding cell lineage development. 

#### 2.2.1. Basics of the Quail-Chick Chimeric System

**Figure 2:**
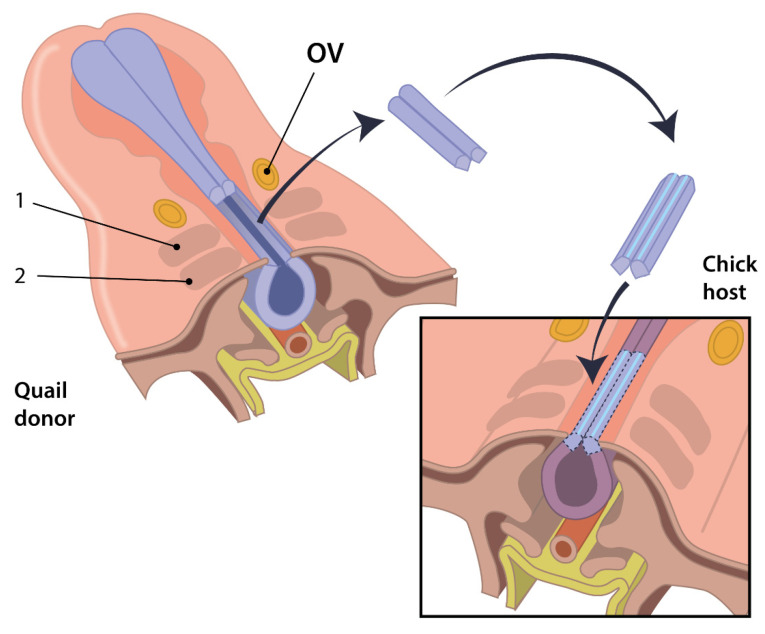
The quail-chick chimeric system. A schematic representation of heterospecific transplantation of the neural crest tissue common to chimeric lineage tracing. The dorsal part of the neural tube of the quail, containing the neural crest cells, is excised and interchanged into a chick host of a comparable embryonic stage at the same locus. 1: somite 1, 2: somite 2, OV: otic vesicle. Image obtained with permission from TP Kelder & R Vincente-Steijn et al. [48].

The quail chick chimera system was devised in 1969 [60,61,62]. In the quail-chick chimera system a subset of cells derived from quail, i.e., cells of the neural fold, are transplanted in stage-matched chick embryos (or vice versa) (Figure 2). As a result, the quail derived cells develop into functional tissues resulting in viable chimeric quail-chick offspring. The quail-chick marker system relies on the differences in heterochromatic DNA in the nuclei of chick and quail. With the use of a nuclear Feulgen staining or a donor specific antibody [63] distinction could be made between host and donor cells, allowing researchers to study embryonic development at later stages as compared to vital dye labelling. In addition to the quail-chic chimeric system, xenopus hybrids were also used to study early stages of embryonic development [64]. 

#### 2.2.2. Advantages of the Quail-Chick Chimeric System

The quail-chick chimera system allows for cell lineage tracing using the genetic footprint of the host cells. As a result, this method of lineage tracing results in a permanent change through which chimeric models overcome the limitation of vital dye dilution mentioned previously. Therefore, chimeric systems allow researchers to examine a specific cell population for longer time periods as compared to injecting cells with vital dyes. Moreover, whilst vital dyes may suffer from dye leakage during injection, chimeric models have no such issues. Another advantage of chimeric models is that it enabled researchers to perform in-vitro cell manipulation prior to transplantation.

#### 2.2.3. Disadvantages of the Quail-Chick Chimeric System

Similar to vital dyes, chimeric models require physical access to an embryo favouring oviparous vertebrates over mammalian embryos. Nevertheless, lineage tracing through cell transplantation has often been applied in mice studies examining multilineage differentiation through bone marrow transplantation in irradiated mice but also to study early mammalian development through injection of cells in the developing morula [65]. Moreover, with the recent developments in stem cell biology the study of heart development using mice-rat chimeric models could lead to new insights [66]. In contrast to vital dye injections, in chimeric models a graft versus host rejection may occur in postnatal studies. Another challenge when making interspecies grafts is the need to perform microsurgery on two stage-matched embryos increasing the experimental complexity. Moreover, chimeric models are generally best used when cells are transplanted superficially, such is the case with cells of the spinal cord or epicardium. Transplantation of difficult to reach mesodermal cells is likely to complicate experimental reproducibility.

#### 2.2.4. The Quail-Chick Chimeric System and Insight into OFT Development

Chimeric models like the chick-quail system were paramount for understanding the role of NCCs in cardiac development. Le Lievre and LeDouarin showed that NCCs formed the tunica media of the great arteries [67]. Kirby and Steward used quail-chick chimeras to observe migration of NCCs into the heart, contributing to the aortopulmonary septum and showed how ablation of the NCC population resulted in persistent truncus arteriosus [34]. Moreover, transplantation studies using chimeric models made the initial observation of cellular pluripotency because quail NCCs could expand into subsets of totipotent and pluripotent progenitors [68]. The quail-chick chimera system was also used to determine that neural crest cells were contributing to the formation of semilunar valves [69] as well as to elastogenesis in the developing cardiovascular system [70]. A role for NCCs in coronary artery formation was demonstrated by Arima et al. which found NCCs contributing to heart development and smooth muscle heterogeneity within a coronary artery using the quail-chick chimeric model [71].

### 2.3. Genetic Lineage Tracing

A widely used tool in developmental biology is based on site-specific recombination within the genome. Most mouse models developed for genetic lineage tracing utilise the P1 Bacteriophage derived enzyme Cre-recombinase, which recognises a 34-bp sequence called the *loxP* site, and induces recombination between pairs of these sites [72]. The Cre-*loxP* system, combined with the ability to knock-in sequences at specific sites in the genome has been used to study the origin of the developing OFT.

#### 2.3.1. Basics of Genetic Lineage Tracing 

**Figure 3:**
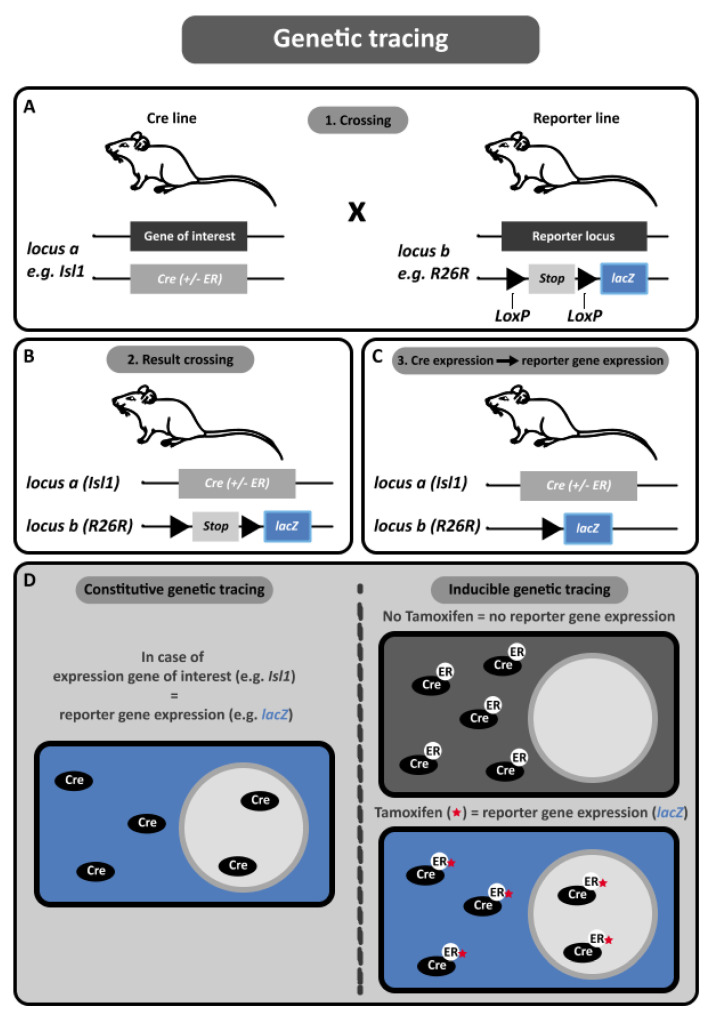
Genetic lineage tracing. (**A**) Genetic lineage tracing can be achieved using the Cre-*LoxP* system. Upon expression, the Cre recombinase specifically targets *LoxP* DNA motives and allows for excision of DNA elements floxed by *LoxP* sites. The reporter line contains a stop-cassette inhibiting expression of the reporter gene in the absence of Cre. (**B**) Crossing the reporter line with the Cre line results in offspring carrying both constructs. (**C**) Cells expressing the gene of interest will express the Cre-recombinase. Cre will then permanently remove the stop-cassette and thus allow expression of the reporter gene, resulting in cell lineage labelling. (**D**) Modified Cre-recombinases can be used to allow for more temporal control in genetic labelling. Cre-ER variants can prevent Cre-recombinase activity by restricting mobility to the cells cytoplasm in absence of Tamoxifen. Upon stimulation of Tamoxifen-Cre activity is restored resulting in an inducible system for genetic lineage tracing.

To use the Cre-*loxP* system for genetic tracing, several steps are necessary (Figure 3). The basis of the Cre-*loxP* system is crossing a Cre transgenic mouse strain with a mouse in which a reporter gene is flanked by *LoxP* sequences (“floxed”). First, the sequence coding for Cre-recombinase is knocked into the locus of the gene of interest, for example Isl1. The next step is crossing the Isl1^Cre^ mouse line with a reporter line (Figure 3A). The genetic tracing experiments in this review were mostly performed in reporter lines that utilise the Rosa26 locus (R26R), but different loci (e.g., Gata4 [73]) have also been used in other studies. Important characteristics of a potential reporter locus are ubiquitous expression of the gene and viability and fertility of the mouse strain after homozygous inactivation of the reporter locus by insertion of a reporter gene, such as *LacZ* or a single or multiple fluorescent proteins. To control expression of the reporter gene, a floxed sequence preventing transcriptional read-through (also referred to as a “stop-cassette”) is placed before the reporter gene sequence (Figure 3A). 

Crossing the floxed reporter line with the cell type specific Cre line results in offspring carrying both constructs (Figure 3B). Cells expressing the gene of interest will produce Cre-recombinase, which will result in permanent deletion of the stop-cassette and expression of the reporter gene (Figure 3C). Since expression is under control of the ubiquitous Rosa26 promoter, these cells and their progeny will retain reporter gene expression, thereby enabling tracing of these cells over a long period of time. The above-described method is referred to as “constitutive” genetic tracing in this review (Figure 3D). 

A variation of genetic tracing, referred to as “*inducible*” genetic tracing, enables temporal control of recombination (Figure 3D). This system is based on fusion of a mutated ligand-binding domain (LBD) of the human estrogen receptor (ER) and Cre-recombinase. The resulting protein is only sensitive to the anti-estrogen Tamoxifen, not to the endogenously present estradiol [74]. The fusion gene is inserted in the locus of the gene of interest, which results in production of the fusion protein upon expression of the gene of interest. The Cre fusion protein is located in the cytoplasm and is unable to enter the nucleus, due to the presence of the mutated LBD. Administration of Tamoxifen results in binding to the LBD, which enables translocation of Cre-recombinase to the nucleus, where it induces gene specific recombination [75]. Thus, recombination can only occur after administration of Tamoxifen, resulting in temporal control of lineage labelling (Figure 3D).

In 2007, multicolour lineage tracing using the Brainbow labelling cassettes was introduced to the genetic lineage tracing toolset [76]. The Brainbow cassettes (Brainbow-1,2 and 3.2) are genetic labelling constructs exploiting the Cre-*loxP* system to allow for stochastic recombination events to obtain single cell resolution lineage labelling. This method is based on combining multiple fluorescent reporter gene sequences in the Brainbow cassettes flanked by a combination of cis- and trans-*loxP* sites. Upon Cre expression, driven by the gene of interest, the fluorescent reporter genes randomly change configuration within the cassette affecting expression of the cell marker. Because multiple copies of fluorescent reporters are embedded within the Brainbow cassettes (i.e., CFP, YFP, RFP) it allows for multiple fluorescent signatures related to the combination of reporters being stochastically expressed after recombination (i.e., Expression of a CFP-CFP-CFP combination would allow for a “Blue” signal, whilst a RFP-RFP-CFP combination would result in a “Magenta” signal). Combining multicolour lineage tracing with tamoxifen induction can result in a multicolour lineage tracing model with temporal control. 

#### 2.3.2. Advantages of Genetic Tracing

The most important advantage of genetic tracing is the ability to visualise the complete heritage of a progenitor population driven by a single marker gene of interest. Combination of these results with previous known expression patterns (e.g., based on immunohistochemical staining patterns) allows for the reconstruction a genetic fate map during development of the OFT. 

Furthermore, genetic lineage tracing is non-invasive, as long as insertion of the sequence for Cre does not impair normal development of the embryo. This is an important advantage as compared to performing prospective tracing with microinjection of vital dyes or viral constructs (see Section 2.1.3).

Inducible genetic tracing has an important advantage over constitutive genetic tracing, as it allows for temporal control of Cre expression. Constitutive genetic tracing is solely dependent on the natural timing of Cre expression related to the driving promotor. Expression of the gene of interest at any given (and frequently unknown) time point will result in reporter gene expression (if the level of Cre expression is sufficient to induce reporter gene expression, see below). With inducible tracing, reporter gene expression will only occur in the presence of Tamoxifen [74].

#### 2.3.3. Disadvantages of Genetic Tracing

Reporter gene expression in the tissue or structure of interest can result from several scenarios. In the ideal situation, it can show that at time point 0, a certain structure with known expression of the gene of interest is a progenitor for the structure with reporter gene expression at a later time point. However, a possible pitfall can arise from interference resulting from aspecific lineage markers. In scenarios when a gene of interest gives rise to multiple progenitor populations of different origin, interpretation of developing structures would be troubled, which could lead to an inaccurate conclusion regarding the lineage developmental contribution. This difficulty in interpretation of genetic tracing experiments was demonstrated by constitutive genetic tracing of *Tbx18*. *Tbx18* is commonly used as a marker for the sinus venosus and epicardium. Constitutive genetic tracing was performed with a Tbx18*^Cre^* in the R26R*^L^^acZ^* background and reporter gene expression was found in myocardial cells, which led to the conclusion that these cardiomyocytes derived from epicardial cells [77]. However, later experiments showed active expression of *Tbx18* in cardiomyocytes, which was put forward as an explanation for the observed reporter gene expression [78]. Using constitutive genetic tracing, reporter gene expression in cells shows that either these cells have expressed or still actively express the gene of interest.

Even though timing is far more accurately determined using inducible genetic tracing, exact timing of recombination and reporter gene expression remains difficult. It was shown that after intraperitoneal injection of Tamoxifen at E8.5 in pregnant dams, the first signs of *LacZ* activity in the embryos were seen 6 h after injection, with an increase in expression at 12, 24 and 48 h [79]. Analysis of reporter gene expression after Tamoxifen injection therefore is dependent on the duration of Tamoxifen exposure, which complicates exact timing. Furthermore, the dosage of Tamoxifen has also been shown to influence reporter gene expression, with higher doses resulting in reporter gene expression in more cells [79]. Recent findings also revealed that the basal activity of Tamoxifen induced Cre (CreERT2) can be sufficient to induce genetic recombination even in the absence of tamoxifen induction [80]. Depending on the reporter line this may result in aspecific cell labelling [80]. This shows that analysis of reporter gene expression and interpretation of these results can be a challenge and might require further substantiation to guarantee efficacy.

The complete absence of reporter gene expression is also difficult to interpret as this can be caused by absence of expression of the gene of interest, or absence of recombination due to sub-threshold levels of Cre expression, not sufficient to induce reporter gene expression. 

Recent studies might raise questions regarding the accuracy of using Cre driven genes as a reliable cell lineage marker. In genetic tracing experiments, interpreting specific gene expression can be problematic, but a far more complex problem arises in case of lack of reporter gene expression. A large number of genetic tracing experiments utilise the *R26R* locus, which is also the most commonly used reporter locus described in the current review. Genetic tracing experiments comparing the *R26R* locus with a *Gata4* based reporter system showed differences in reporter gene expression between the two lines [73]. Constitutive genetic tracing of *Isl1* and *Nkx2-5* (both genes are discussed in more detail below) showed more extensive reporter gene expression in the *Gata4* reporter line, indicating that the *Gata4* locus was more sensitive to recombination then the *R26R* locus [73]. The difference in reporter gene expression shows that different reporter strains have different thresholds for reporter gene expression, resulting in different conclusions based on the reporter used. An example of the importance of this is shown by reassessment of the fate of *Isl1*+ progenitors [73]. This gene is commonly used as marker for the SHF [81,82]. However, the expanded pattern of reporter gene expression in the *Gata4* reporter line showed that nearly all cells (including the LV, which is commonly described as being derived from the FHF) of the heart derive from progenitor cells that did express *Isl1*, albeit perhaps at a low level for a brief period. This suggest that Isl1 is either not specific enough or not reliable as a SHF marker [73]. In this case, mRNA [83] and protein [40] expression of Isl1 in the cardiac crescent, classically considered to be a FHF structure, further support these observations. 

**Figure 4:**
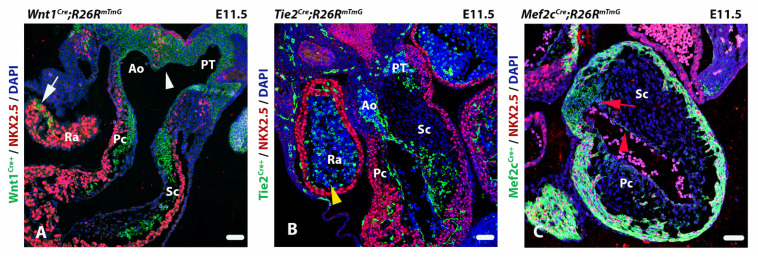
Transversal sections of immunofluorescent outflow tracts show challenges with genetic lineage tracing. (**A**) A *Wnt1^Cre^; R26R^mTmG^* embryo showing neural crest cell derived cells (green). Interestingly, *Wnt1* derived cells with NKX2.5 stained nuclei can also be observed at the so-called flow divider [84], in front of the primitive foregut which is the central part of the second heart filed contribution (white arrowhead) as well as in the atrial myocardium (white arrow). (**B**) a *Tie2^Cre^; R26R^mTmG^* embryo showing endothelial derived cells (green). Endothelial cells undergoing epithelial to mesenchymal transition give rise to cells within the septal and parietal cushions. Cells derived from myeloid lineages also express *Tie2* resulting in lineage positive blood cells (yellow arrowhead). (**C**) A *Mef2c^Cre^*; *R26R^mTmG^* embryo showing second heart field derived cells (green). Cre positive endothelial cells can also be found lining the heart (red arrowhead). *Mef2c* derived cells also contribute directly to the septal cushion (red arrow). Ra: Right atrium, Ao: Aorta, PT: Pulmonary trunk, Pc: Parietal cushion, Sc: Septal cushion. Scalebar: 50 µm.

Therefore, when performing constitutive or inducible genetic tracing experiments, one should be cautious with interpretation of the results (Figure 4 illustrates this in more detail). In constitutive genetic tracing, reporter gene expression demonstrates that labelled cells have expressed or still actively express the promotor driven Cre. It does not provide information on the initial source/location of the progenitor cells. With inducible genetic tracing, temporal control of Cre expression is possible. Expression of the reporter gene shows that Cre is present in the labelled cell between the time of Tamoxifen administration and the moment of analysis. Finally, interpretation of negative results in genetic tracing is challenging and results need to be verified by additional tracing techniques and gene/protein expression experiments at sequential stages of development. 

#### 2.3.4. Using Genetic Lineage Tracing to Study OFT Development

Genetic lineage tracing has been applied extensively to identify cell lineages contributing to OFT formation. The early observations of neural crest derived cells contributing to the developing heart were further substantiated using the neural crest specific *Wnt^Cre^* transgenic mouse (Figure 4A) [20]. This model, when crossed with a Cre-specific reporter line, allowed for lineage tracing of NCCs revealing their role in semilunar valve development as well as the cardiac conduction system [85]. The appearance of the OFT cushions and a role for endothelial cells was observed in early studies using electron microscopy [86]. However, the development of the *Tie2^Cre^* transgenic model was essential for the study of endothelial cell lineages in vivo (Figure 4B) [22]. Other genetic markers have been extensively used to define cell lineages such as the *Isl1+* SHF [81,82] and the *Mef2c^Cre^* SHF lineage model (Figure 4C) [87]. Table 1 shows an overview of genetic lineage tracing models that are often used in OFT studies, the morphological structures these lineages contribute to, and the possible conflicts arising from the genetic lineage interpretations.

Recent developments in genetic lineage tracing have introduced a dual genetic tracing system to address the dynamic origins of cardiac valve mesenchyme [88]. Dual genetic tracing allows for the study of multiple cell lineages simultaneously by using multiple site-specific recombinases such as Nigri-*nox* to complement the Cre-*loxP* system [89]. Dual genetic lineage tracing could aid in addressing the specificity concerns of traditional Cre-*loxP* lineage tracing systems.

**Table 1:** An overview of common genetic lineage tracing models used in OFT studies.

Transgenic Mouse Lines	Commonly Used as Cell Lineage Marker for:	Observed Tissue Expression	Possible Lineage Conflicts
*Hcn4^Cre-ert2^*	First heart field [90]	Cardiac conduction system [90] Myocardium [90]	Second heart field
*Hoxa1^Cre^*	Cardiac precursors [91]	Aortopulmonary septum [91] Cardiac conduction system [91] Coronary arteries [91] Endothelial lining [91] Myocardium [91] Semilunar valves [91]	Endothelial Neural Crest Second heart field
*Isl1^Cre^*	Second heart field [81]	Atrioventricular valves [73] Cardiac conduction System [90] Cushion mesenchyme [73] Endocardium [73] Myocardium [92] Proepicardium [93] Semilunar Valves [73]	Endothelial Epicardial First heart field Neural Crest
*Krox20^Cre^*	Neural Crest [94]	Endocardium [94] Semilunar valves [94]	Endothelial
*Mef2c^Cre^*	Second heart field [87]	Ascending aorta [95] Coronary arteries [96] Cushion mesenchyme [95] Endocardium [95] Myocardium [87,95] Semilunar valves [95]	Endothelial Epicardial
*Nkx2-5^Cre^*	First and Second heart field [97]	Ascending aorta [98] Coronary artery [98] Endocardium [73] Epicardium [93] Myocardium [97], Semilunar valves [98]	Endothelial Epicardial
*Pax3^Cre^*	Neural Crest [99]	Aortopulmonary septum [100] Ascending aorta [99] Cushion mesenchyme [100] Semilunar valves [100]	
*Tbx18^Cre^*	Proepicardium/epicardium [77]	Cardiac conduction system [90] Epicardium [77] Myocardium [77]	First heart field Second heart field
*Tbx2^Cre^*	Proepicardium/epicardium [101]	Cardiac conduction system [102] Coronary arteries [101] Epicardium [101] Myocardium [101,103]	First heart field Second heart field
*Tie2^Cre^*	Endothelium [22]	Atrioventricular valves [22,104] Coronary arteries [105] Cushion mesenchyme [22,104] Endocardium [22,104] Hematopoietic cells [106] Semilunar valves [104]	Hematopoietic
*Tnnt2^Cre^*	Myocardium [107]	Ascending aorta [25] Myocardium [25,107] Semilunar valves [25]	
*Wnt11^CreER^*	Cardiac precursors [108]	Endocardium [108] Epicardium [108] Myocardium [108] Semilunar valves [108]	First heart field Endothelial Epicardial Second heart field
*Wnt1^Cre^*	Neural Crest [20]	Aortopulmonary septum [20] Ascending aorta [20] Cardiac conduction system [109] Coronary arteries [71] Cushion mesenchyme [20] Epicardium [110] Myocardium [110,111] Semilunar valves [20]	Epicardial Second heart field
*WT1^Cre^*	Proepicardium/epicardium [112]	Coronary arteries [112] Epicardium [93] Myocardium [93]	Endothelial lineage First heart field Second heart field

### 2.4. Retrospective Clonal Analysis

The aforementioned techniques can all be described as prospective fate mapping techniques. Knowledge on gene and protein expression profile, timing of expression and/or location of progenitors is essential to perform subsequent prospective labelling of this progenitor pool [52]. The following section focuses on retrospective clonal analysis.

#### 2.4.1. Basics of Retrospective Clonal Analysis

**Figure 5:**
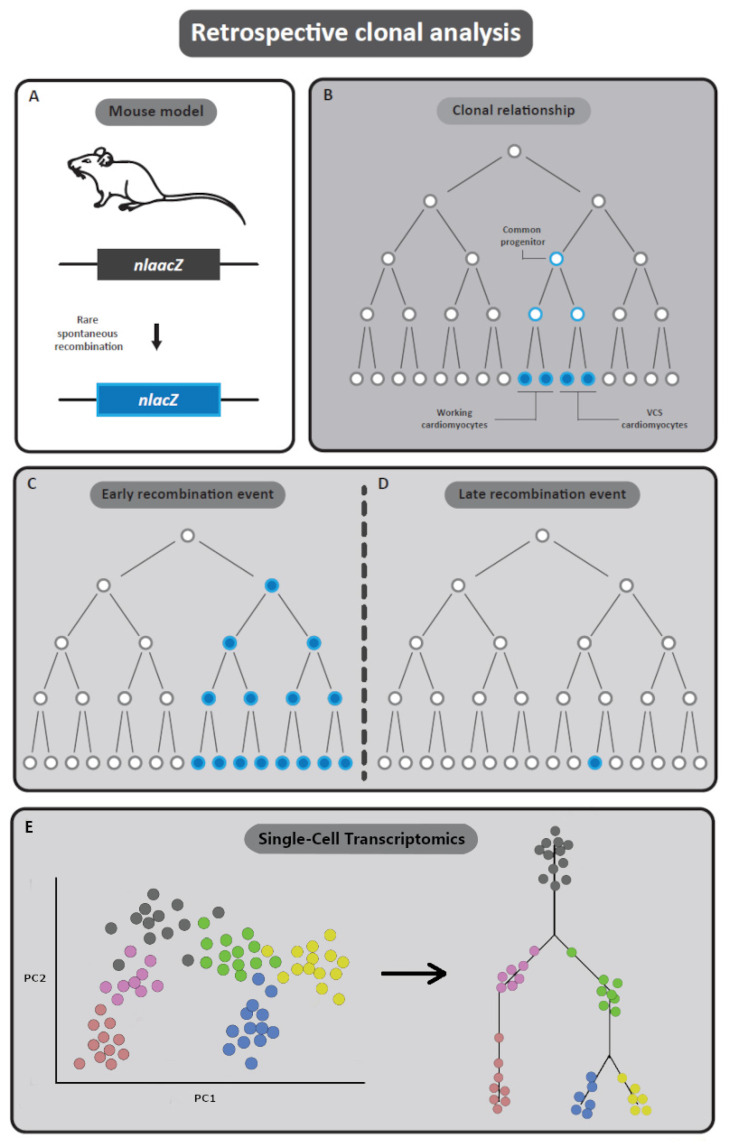
Retrospective lineage tracing. (**A**,**B**) Traditional retrospective lineage tracing exploits spontaneous and rare recombination events. The nlaacZ sequence was designed to be inactive by default but increase the odds of recombination events within the region. Upon spontaneous recombination, β-galactosidase is expressed resulting in a lineage label. (**C**,**D**) Early recombination (i.e., during early stages of development) will give large clusters of cells with reporter gene expression, whilst late spontaneous recombination results in smaller clusters of *LacZ* positive cells. (**E**) Modern retrospective lineage tracing derives lineage hierarchy from single cell transcriptomics data. Principle component analysis (PC) aids in clustering groups of cells based on similarity in gene expression profiles. Follow up algorithms can then produce a phylogenetic lineage tree derived from expressional gradients among cell clusters to reconstruct cell lineage development.

Retrospective clonal analysis is based on infrequent and spontaneous recombination of the *nLaacZ* reporter [113]. This reporter gene encodes the *LacZ* gene, with an inactivating duplication inserted in the reading frame, thereby inhibiting transcription of functional β-galactosidase. A spontaneous and rare recombination event can result in removal of the duplication and subsequent production of β-galactosidase (Figure 5A) [113]. Reporter gene expression can be followed in progeny of cells in which spontaneous recombination of the *nLaacZ* construct occurred [113]. Targeting of this construct to an allele which is highly expressed in cardiac muscle, (e.g., α-cardiac-actin) enables retrospective clonal analysis of cardiac cells, irrespective of gene expression [114,115,116].

Clonal analysis is based on statistical evaluation of the chance that reporter gene expression in different cells is based on separate spontaneous recombination events, or that these different cells are progeny of one recombination event (and thus share a common progenitor during development). A statistical method commonly used is the fluctuation test of Luria and Delbrück [117]. This famous experiment showed that mutations in bacteria occur spontaneously (not induced by selection) and at a constant rate, which was used to formulate a probability distribution [117]. This distribution is used to calculate the probability of one *or* more recombination events, and will therefore determine whether cells showing reporter gene expression are most likely clonally related or are derived from different recombination events (Figure 5B) [113,114,115,118]. Early recombination (i.e., during early stages of development) will give large clusters of cells with reporter gene expression (Figure 5C), while late spontaneous recombination will give smaller clusters of *LacZ* positive cells (Figure 5D). 

Recent studies performing retrospective clonal analysis have been adopting next-generation sequencing techniques to study lineage tracing. Recent advances in single cell sequencing technologies allow transcriptome profiling of thousands of single cells. This allowed for the collection of large datasets detailing cellular expression profiles with unprecedented resolution. Alongside advances in sequencing technologies, computational methods designed to examine lineage trajectory reconstruction based on single-cell transcriptomics data have also evolved [119,120]. Single-cell transcriptomics allows for the investigation of the transcriptional state of thousands of individual single cells. As a result, cell-type diversity in heterogeneous samples can be reliably captured. As cells transition between different states during embryologic development, their expression profiles give insight into their lineage fate and cellular identity. 

If sufficient amounts of cells in these transition states are captured, differentiation trajectories through which tissues are derived can be reconstructed through hierarchical clustering of cells based on the gradient in expressional similarities (Figure 5E). 

#### 2.4.2. Advantages of Retrospective Clonal Analysis

To perform prospective lineage tracing, some basic knowledge on the progenitor population that will be traced is required. This information is not necessary when performing retrospective clonal analysis. Studying whether certain structures or clusters of cells are clonally related is done independent of gene expression or any other preconceived idea of a possible progenitor pool. Retrospective clonal analysis can therefore establish clonal relationships that are less apparent at first sight, such as the clonal relationship between head musculature and cardiomyocytes from the outflow tract and right ventricle [121,122].

Furthermore, as described previously, prospective genetic tracing is hampered by the difficulty to draw conclusions from the absence of reporter gene expression. With the retrospective clonal approach, analysis is based on *LacZ* positive cells. *LacZ* expression in different structures or tissues is mapped and statistical analysis is performed to calculate whether these different tissues or structures are clonally related [113]. Moreover, as with genetic tracing, retrospective clonal analysis is non-invasive. 

When performing retrospective lineage tracing using single-cell sequencing, an advantage is the ability to perform lineage tracing using naturally occurring somatic mutations or copy number variations (CNVs). CNVs can be used as genetic markers to link clones of cells to a common progenitor. This approach allows for lineage tracing without manual labelling or genetic modification. Even though CNVs are relatively uncommon in healthy tissue, they are highly abundant in cancerous tissue and have been used to study tumour evolution [123,124]. Alternative to CNVs is lineage tracing using single-nucleotide variations (SNVs) and genomic insertion/deletions (indels). Both SNVs and Indels are often present in non-coding regions of the genome and have been used for the reconstruction of phylogenetic trees of tumours from bulk DNA [125]. This technique, however, is not limited to tumour lineage analysis as lineage tracing using somatic SNVs in mitochondrial DNA can be performed with any eukaryotic cell [126]. Moreover, combining the genetic variation in non-coding regions of DNA with the RNA expression profiles when performing lineage tracing has recently been successfully demonstrated [127].

#### 2.4.3. Limitations of Retrospective Clonal Analysis

The retrospective approach has several drawbacks. Since data is analysed retrospectively, it is not possible to locate the common progenitor, either in time or space. Therefore, it is not possible to perform further experiments with these progenitors (e.g., characterisation or ablation). Retrospective clonal analysis has to be performed in conjunction with other fate mapping techniques to build the complete lineage tree of a structure or organ. In this sense, the most important advantage of this technique is also its most important drawback. Starting with a certain progenitor pool based on timing, location and/or gene/protein expression profile excludes contributions from other (sometimes unexpected) progenitors but does directly pinpoint a possibly important progenitor pool for the structure/tissue of interest. Furthermore, spontaneous recombination of the *nlaacZ* gene is rare and it is therefore necessary to obtain a large library of embryos with *LacZ* positive clones and as a result, large quantities of embryos or adult mice will have to be analysed. A challenge when performing single cell sequencing for lineage tracing lies in the sparse distribution of SNVs and CNVs within the genome. To achieve confident detection of SNVs, large quantities of reads will have to be generated to achieve adequate sequencing depth. A large portion of the generated data is unsuitable for lineage tracing as these would contain identical sequences. However, enrichment of regions of DNA with greater odds of developing nucleotide variations such as microsatellites could apprehend these challenges. 

#### 2.4.4. Retrospective Clonal Analysis Aimed at the Development of the OFT

Many of the cardiac lineages contributing to OFT development have been found using prospective lineage tracing techniques (Table 1). However, a recent study examining cell populations at the aortic root and pulmonary trunk determined a novel SHF derived cell population called arterial mesothelial cells (AMC) populating a local niche at the base of the great arteries [128]. Whilst AMCs have first been observed as a distinctive cell type using chick-quail chimera’s [129], the study of Lioux et al. used a clonal analysis strategy based on the ubiquitous, low-frequency random recombination of two independent reporters to distinguish this population from the frequency of colour matching between labelled cells in different clusters or different cell types within single clusters [130].This approach allowed for an unbiased characterisation of the fate of SHF precursors at the base of the great arteries and detailed the contribution of SHF to the coronary lymphatic vasculature [128]. Moreover, another study examining the cardiac outflow tract using single cell RNA sequencing (scRNA-seq) found convergent development of the vascular smooth muscle cell lineage suggesting a method of myocardial-to-VSMC differentiation or mesenchymal-to-VSMC differentiation [131].

## 3. New Technologies for Lineage Tracing in OFT Development

Lineage tracing has contributed greatly to our understanding of OFT development. Nevertheless, there is still much to be learned regarding multifactorial CHDs. To advance our understanding of phenotypical variation, research efforts should be aimed at understanding the relation between cell lineage and cellular identity. The methods previously described in this review are inadequate to address such issues, as this would require detailed description of the variation among single cells within a lineage population. The introduction of novel analysis methods which combine lineage tracing and single cell analysis could provide new opportunities to examine cell lineage plasticity and phenotypical variation in CHD. These methods will be discussed in the following section.

### 3.1. Spatially Resolved Transcriptomics

Current lineage tracing techniques, such as genetic lineage tracing, rely primarily on spatial information to reconstruct OFT development over multiple embryonic stages. The tracking of cell lineage movements can give insight into the environment in which these cells function. However, evaluating the internal cellular state of these cells within such environments can be challenging. Single cell RNA sequencing (scRNA-seq) is a technique which can be used to examine a cell’s innerworkings because it allows for quantification of all the RNA molecules within a single cell. This cellular transcriptome can be used to examine gene expression patterns and to make cell type specific expression profiles. By collecting expression profiles of multiple cells of the same cell type, it is possible to examine the cellular variation in gene expression within a specific cell population. This allows researchers to evaluate the variation in cell states within a particular cell lineage, and reconstruct subclusters structural to a cell lineage population. Whilst scRNA-seq can be used to obtain genome-wide expression data of cells allowing for unprecedented cell type identification, it does not provide any spatial resolution to determine where these cells were located within the embryo. As a result, experiments using scRNA-seq to identify new cell types and cell states still require the use of visualisation techniques such as in-situ hybridisation using the genetic markers found during sequencing. However, in-situ hybridisation can only provide spatial information regarding a few genes at any given moment. Tomo-seq is a technique which combines genome-wide expression data with spatial information [132,133]. This is achieved by performing RNA-seq on individual cryosections of an embryo or tissue allowing the generation of genome-wide spatial expression maps in 3D. By sequencing sequential tissue sections, spatial information regarding the head to tail axis can be maintained. As a result, gene expression data can be projected with respect to the head to tail axis. Performing this approach using multiple axis (dorsal-ventral, right-left) allows for the reconstruction of gene expression patterns in 3D in the developing embryo [133]. Although Tomo-seq allows for 3D expression pattern analysis, one limitation is that it is unable to provide single cell resolution in 3D. An alternative method called transcriptome in vivo analysis (TIVA) does allow for single cell sequencing whilst maintaining single cell spatial resolution. TIVA is a noninvasive tool enabling capture of mRNA from single spatially defined cells in living and intact tissue for transcriptome analysis [134]. This is achieved by addition of a cell permeable TIVA tag to the tissue sample which upon photo activation is able to capture cellular mRNA. This technique is an excellent advance over more traditional laser capture techniques or pipette cell isolation adapted for scRNA-seq due to the non-invasive design. The limited throughput, however, might restrict explorative research to specific cases in which the number of cells of interest might be relatively low. A practical example in which TIVA might prove very valuable for OFT studies, is to examine the properties of valvular interstitial cells (VICs) contributing to the aortic and pulmonary valves. VICs may derive from multiple cell lineages such as SHF, NCC or endothelial lineages and VIC dysfunction has been suggested to underly valvular diseases such as calcification [135]. TIVA could enable researchers to examine how cell lineage background influence VIC formation and how cell lineage deficiencies could relate to valvular disease by assigning a spatial criterium to scRNA-seq.

### 3.2. Lineage Tracing through DNA Barcoding

DNA-barcoding technologies are another potential tool for lineage tracing. Lineage tracing using DNA barcoding is a technique that combines aspects of both prospective and retrospective lineage tracing. Similar to genetic lineage tracing using fluorescent reporters, DNA-barcoding technologies introduce genetic modifications into progenitor cells to study cell lineage development. However, whilst lineage tracing using fluorescent reporters is limited to only a number of reporter genes, DNA barcodes allow for more complex labelling methods. Techniques such as scGESTALT [136], ScarTrace [137] and LINNAEUS [138] have demonstrated how the CRISPR-Cas9 system could be used to generate genetic barcodes to perform whole-organism lineage tracing in zebrafish embryos. The CRISPR-Cas9 system allows genomic editing on a specific DNA locus determined by the supplied guide RNA. In the absence of a template for homologous repair, Cas9 may introduce short DNA insertions or deletions at the targeted site [139]. These genetic aberrations, which vary in size and position, can then be used as a genetic barcoded label to evaluate a cell’s offspring. If the guide RNA was designed specific to a region within a constitutive expressed gene, then scRNA-seq can capture the barcode within the transcriptome of a cell. Since the process of barcoding is randomly determined, a cell’s lineage can only be evaluated after single cell sequencing. This can be achieved through computational reconstruction of full lineage trees from the single cell level. Even though these methods still rely on microinjection of Cas9 and guide RNAs in an early stage zebrafish embryos, DNA-barcoding has also been achieved using retroviral integration [140] or polylox recombination [141] in mice. For an in dept comparison of barcoding techniques, the reader is referred to a recent review of Wagner and Klein [142]. Lineage tracing using barcodes could be an excellent method to examine cell lineage commitment because it allows to retrospectively evaluate cell lineage branching events. The differences in lineage trajectories among FHF and SHF lineages would be an interesting topic to be evaluated using such methods. Moreover, if barcoding events are temporally restricted and multiple stages of embryonic development are analysed, then it could enable reconstruction of all cell lineage trajectories contributing to heart development simultaneously. Genetic mutations or epigenetic influences affecting cellular identity could also be examined as these would influence the relative distributions among (sub)populations of cell lineages. As such, these methods could aim to elucidate the relationship between phenotypical variation and cell lineage variation.

### 3.3. Multi-Omics Lineage Tracing

Whilst barcoding techniques for lineage tracing require aspects of a prospective approach to lineage tracing, recent technological developments using a multi-omics sequencing approach for lineage tracing could allow for complete retrospective lineage tracing. RETrace is an example of a recent lineage tracing technique which captures both DNA microsatellite loci and methylation-informative cytosines from a single cell in order to simultaneously characterise both lineage and cell type [143]. The utilisation of microsatellite regions as markers for retrospective lineage tracing was successfully demonstrated at single cell level [144]. However, enabling DNA methylation profiling of the same cell allows for better cell characterisation than either method alone. Even though still in early development, a multi-omics approach to lineage tracing would ideally combine the cell lineage identification capability using natural occurring CNV and SNVs captured using whole genome sequencing whilst allowing for accurate cell type identification through gene expression analysis using scRNA-seq and also include epigenetic profiling techniques such as methylome analysis [145] or include histone signatures [146] to investigate cellular identity and cell lineage dynamics. Multi-omics lineage tracing would enable researchers to investigate the relations between cellular variation, epigenetic modification and disease susceptibility related to CHD. This knowledge could potentially translate to novel methods of treatment and therapy aimed at influencing the epigenome of patients.

### 3.4. Reference Maps for Lineage Tracing

The large amounts of data derived from lineage tracing using transcriptomics are likely to extent the depth at which sub-lineages and cell states will be defined. To facilitate accurate definitions of such sub lineages and intermediate cell states, large quantities of single cell data could be combined. A project called the Human Cell Atlas (HCA) aims to characterise all human cell types using scRNA-seq and single cell transposase accessible chromatin sequencing (scATAC-seq), and combine it with data regarding cell lineage, location and cell states [147]. The scope extents to unify single cell sequencing data towards common reference models similar to the efforts of the Human Genome Project [148]. Having the infrastructure to navigate cell lineage development will greatly contribute to the understanding of human health and treatment of disease. (For more information regarding the HCA project, the reader is referred to the HCA white paper [149]). Initiatives such as these could allow for the comparison of many cell lineage populations among the various types of CHD to determine key processes critical to human cardiovascular development.

## 4. Future Perspectives, Combining Old Strengths with New Technologies

With the advances in lineage tracing, new insights regarding cell fate came forth and the role of prospective lineage tracing paved the way for modern developmental biology. In this review we describe the use of lineage tracing techniques and their limitations with respect to OFT development. The recent technological methods described in the final section of this review have the potential to allow more fateful reconstruction of lineage dynamics than previous methods.

The development of next-generation sequencing technology has enabled new and exciting research opportunities for lineage tracing experiments. From our perspective we expect that results obtained from retrospective lineage tracing using naturally occurring mutations and single cell transcriptomics will challenge our current definitions of cell lineages and advance our understanding of cellular dynamics and lineage plasticity. Assuming technological improvements will continue to be developed in the coming years, we can expect an integration of (epi)genetics signatures with single cell sequencing to aid with tracing cellular heritage. Continued development and a multi-omics approach will allow future research to expand the scope of cellular identity and resolve the mechanisms explaining congenital variation in more detail and with better accuracy than current available methods. As a result, a multi-omics approach will help to elucidate the mechanisms for normal OFT development and aid in understanding the pathophysiological pathways leading to the multivariate phenotypes common to OFT diseases. As sequencing techniques become more and more accessible, multi-omics sequencing approaches combined with single cell resolution will become the new frontier for future lineage tracing studies.

## Abbreviations

AMC	arterial mesothelial cells
SHF	second heart field
AVC	atrioventricular canal
SNV	single nucleotide variation
CHD	congenital heart disease
TIVA	transcriptome in vivo analysis
CNV	copy number variations
VIC	valvular interstitial cell
E	embryonic day
ER	estrogen receptor
FHF	first heart field
GFP	green fluorescent protein
HH	Hamburger and Hamilton
HCA	Human Cell Atlas
LBD	ligand-binding domain
mT/mG	membrane-bound Tomato/membrane-bound GFP
NCCs	neural crest cells
OFT	outflow tract
PHT	primary heart tube
R26R	Rosa26 reporter
scRNA-seq	single cell RNA sequencing
